# The Genes of *CYP*, *ZEP*, and *CCD1/4* Play an Important Role in Controlling Carotenoid and Aroma Volatile Apocarotenoid Accumulation of Apricot Fruit

**DOI:** 10.3389/fpls.2020.607715

**Published:** 2020-12-18

**Authors:** Wanpeng Xi, Lina Zhang, Shengyu Liu, Guohua Zhao

**Affiliations:** ^1^College of Food Science, Southwest University, Chongqing, China; ^2^College of Horticulture and Landscape Architecture, Southwest University, Chongqing, China

**Keywords:** apricot, carotenoid, apocarotenoid, color, aroma, ripening

## Abstract

Carotenoids are important coloration molecules and indispensable component of the human diet. And these compounds confer most of the apricot fruit yellow or orange color. In China, fruit of some apricot cultivar present light-yellow color but strong flowery flavor, however, the chemical mechanism remains unknown. Here, carotenoids and aroma volatile apocarotenoids (AVAs) in three skin types of apricot cultivars (orange, yellow, and light-yellow skinned) were determined by HPLC and GC-MS, respectively. And the transcript levels of carotenogenic genes were analyzed by qRT-PCR. The orange-skinned cultivars “Hongyu” and “Danxing” fruit presented the most abundant total carotenoid, β-carotene and specific α-carotene contents, and β-carotene (52–77%) increased to become the dominant carotenoid during fruit ripening. The transcript levels of lycopene β-cyclase (*LCYb*) and β-carotene hydroxylase (*CHYb*) sharply increased during ripening. The yellow-skinned cultivars “Sulian No. 2” and “Akeyaleke” fruit contained lower levels of total carotenoids and β-carotene but were rich in phytoene. The light-yellow coloration of “Baixing” and “Luntaixiaobaixing” fruit was attributed to low amounts of total carotenoids, lutein, and neoxanthin and an absence of β-cryptoxanthin, but high level of aroma volatile apocarotenoids (AVAs) such as β-ionone were detected in these cultivars fruit, accompanied by low transcript levels of carotene hydroxylase (*CYP*) and zeaxanthin epoxidase (*ZEP*) but high levels of carotenoid cleavage dioxygenase 1 (*CCD1*) and *CCD4*. Correlation analysis showed that the expression level of *CCD1* negatively correlated with carotenoid accumulation but positively with AVAs production. These collected results suggest that both carotenoid biosynthesis and degradation are important for apricot coloration and aroma formation. CYP, ZEP, CCD1, and CCD4 may be the key regulation points for carotenoid and AVAs accumulation in apricot fruit, which provide important targets for quality-oriented molecular breeding.

## Introduction

Carotenoids are pigments that play a major role in the protection of plants against photooxidative processes. They are efficient antioxidants that scavenge singlet molecular oxygen and peroxyl radicals. In humans, carotenoids are part of the antioxidant defense system (Spirt et al., 2010) and include the vitamin A precursor β-carotene, which can enhance immunity and reduce the incidence of various diseases, such as cancer and cardiovascular disease (Fraser and Bramley, 2004). In addition to protecting plants and improving human health, carotenoids confer an attractive visual appearance of plants. The accumulation of carotenoids results in red, yellow, and orange color in plants (Tanaka et al., 2008). Therefore, carotenoids represent a double index of plant appearance and an indispensable component of the human diet (Saini et al., 2015). Based the point, increasing attention is being paid to the biosynthesis and metabolism of carotenoids in horticultural crops (Enfissi et al., 2017).

Apricot (*Prunus armeniaca* L.) is a very important fruit tree within Rosaceae, and its fruits are appreciated by consumers for their characteristic color, flavor, and juiciness, which are strongly related to the variety and ripening process (Botondi et al., 2003); these fruits are also important sources of fiber, provitamin A, vitamins, and phenolic compounds (Erdogan-Orhan and Kartal, 2011; Melgarejo et al., 2014). As one of the world’s sources of apricot, China has rich apricot germplasm resources, and many cultivars with diverse quality traits exist (Zhang et al., 2019). These apricots present colorful appearance that ranges from light-yellow to yellow or orange, and the fruits of some cultivars exhibit a red blush against the yellow or orange background. These different resource types are ideal natural materials for studying the mechanisms of coloration and colorant metabolism in fruit.

In recent years, carotenoid metabolism has been investigated in many horticultural crops (Yuan et al., 2015), and the biosynthesis pathways have been well established. Carotenoids are biosynthesized in plastids and stored in plant cells. The biosynthesis process starts with the condensation of two geranylgeranyl pyrophosphate (GGPP) molecules by phytoene synthase (PSY) to form colorless phytoene as a 15-*cis* isomer, after which phytoene is converted into red lycopene via a series of desaturation and isomerization steps. Next, all-*trans-*lycopene is cyclized to form orange carotenoid molecules and ultimately hydroxylated to yield yellow xanthophyll (Nisar et al., 2015). Carotenoids can be oxidized to apocarotenoids through symmetrical 9–10 and 9′–10′ cleavage by carotenoid cleavage dioxygenases (CCDs) (McQuinn et al., 2015). The carotenoids existing in many fruits have been characterized, and the profile depends on many factors, such as environmental conditions, the cultivar and the fruit ripening process, and many studies have suggested that the dynamic profile of carotenoids during ripening serves as a basis for understanding their molecular mechanism (Luan et al., 2020).

Previous studies have shown that the yellow or orange background skin coloration of apricots is determined by carotenoids (Le Bourvellec et al., 2018) and that carotenoid accumulation in apricots is regulated by ethylene (Marty et al., 2005). However, the molecular basis of carotenoid accumulation during apricot fruit development has yet to be reported, and the differences between various cultivars remains elusive. In this study, we compared the profile of carotenoid and aroma volatile apocarotenoids in three skin types of apricot cultivar peels and flesh and investigated the pattern of 11 genes involved in carotenogenesis during fruit development and ripening. Our results reveal new insight into apricot coloration and aroma formation, and provide important information for understanding carotenoid metabolism and regulation in apricot fruit.

## Materials and Methods

### Fruit Materials

Apricots were collected from the orchard of the National Fruit Tree Germplasm Repository, Academy of Xinjiang Agricultural Sciences, Luntai, Xinjiang, China (45″191 N, 86″031 E). All experimental trees were planted at a 3–4 m spacing in 1998 in rows in a north-south orientation. The same tree shape (open-center shape), fertilization management, and pest control were used for all experimental trees. Experimental design was a singletree plot complete randomized design with 10 individual trees as replications for each cultivar. From April to July 2017, the fruits of six cultivars (“Hongyu,” HY, with a red peel and orange flesh; “Danxing,” DX, with a red peel and orange flesh; “Sulian No. 2,” SL, with an orange-red peel and yellow flesh; “Akeyaleke,” AK, with an orange-red peel and yellow flesh; “Baixing,” BX, with a light-yellow peel and flesh; “Luntaixiaobaixing,” LT, with a light-yellow peel and flesh) were picked at the fruitlet (F, 21 days after blossoming, DAB), enlargement (E, 32 DAB for “HY” and “DX,” 27 DAB for “SL,” “AK,” “BX,” and “LT”), turning (T, 61 DAB for “HY,” “SL” and “BX,” and 56 DAB, 58 DAB and 57 DAB for “DX,” “AK,” and “LT,” respectively), commercial maturation (CM, 74 DAB for “HY,” “DX,” “SL” and “AK,” and 88 DAB for “BX,” 65 DAB for “LT”), and fully ripe (FR, 82 DAB for “HY” and “DX,” 85 DAB for “SL” and “AK,” 91 DAB for “BX,” 74 DAB for “LT”) stages. After being picked, the fruits were immediately transported to the laboratory, and fruits without mechanical damage or pests were used. Each replicate consisted of 50 fruits, among which 20 fruits were used to determine the basic fruit quality index, and the others were divided into peel and flesh tissues, which were cut into small cubes, immediately frozen in liquid nitrogen and stored at −80°C for later analysis. Three replicates were used for each sample.

### Equations Determination of the Basic Fruit Quality Index

The firmness of the flesh was measured with a hardness tester (Model: HL-300, Xianlin Non Detection Device Co., Ltd., Nanjing, China) with an 8 mm probe. A handheld digital refractometer (B32T Brix Meter, Guangzhou Ruiqi Trading Co., Ltd., Guangdong, China) was used to determine the total soluble solid (TSS) content of the fruit. Titratable acids (TA) were determined by the NaOH titration method. The titratable acidity was calculated with the following formula: TA [mmol/100(mL)] = [(c × V_1_)/V_0_] × [100/V] × 100, where c is the molar concentration of the NaOH standard solution, which was 0.1 mol/L; V_1_ is the volume of NaOH solution consumed by the titration; V_0_ is the volume of the sample used for titration, which was 30 mL; V is the volume of the juice, which was 10 mL; and 100 is the volume after the juice is diluted. The titratable acidity of fruit juice is often expressed as the acid percentage, calculated according to the following formula: TA (%) = [(c × V_1_ × k)/V_0_] × [100/V] × 100. The main organic acid in apricot is malic acid, and the conversion factor, k, of malic acid is 0.067. Fruit color at different developmental stages was measured with a Hunter Mini Scanning Colorimeter (Hunter Associates Laboratory, Inc., Reston, VA, United States), and the color index CCI (citrus color index) was calculated with the following formula: CCI = 1000 × a^∗^/(L^∗^ × b^∗^).

### Carotenoid Extraction and Quantification

The extraction and quantification of carotenoids was carried out according to our previous study (Zheng et al., 2016). Twenty grams of flesh or eight grams of peel was dissolved in 50 mL of extraction solvent (hexane/acetone/ethanol, 50:25:25). After standing for 30 min, the mixtures were centrifuged for 5 min at 6500 rpm. The top colored layer of hexane was recovered and transferred to a volumetric flask. The hexane extract was blown dry with nitrogen and dissolved in 2 mL of methyl tert-butyl ether (MTBE). Then, the solution was transferred to 2 mL of 10% methanol/potassium hydroxide. The mixture was allowed to stand and separate in a separatory funnel, then rinsed twice with water and once with 0.1% butylhydroxytoluene (BHT)/MTBE. The rinse solution was transferred to a brown bottle and dried with nitrogen. After drying, 2 mL methanol/acetone (2:1) was added to the brown bottle for dissolution and filtering with a 0.22 m filter membrane, and the resultant sample was prepared for carotenoid determination.

Carotenoids were separated and detected by HPLC (Waters, Milford, MA, United States) with C30 chromatography columns (250 mm × 4.6 mm, 5 μm, YMC, Wilmington, NC, United States) and photodiode array detector. The mobile phase flow rate was 1 mL/min, the column temperature was 25°C, the detection wavelength was 288 nm, and the injection volume was 20 μL. The mobile phase consisted of methanol, MTBE, and water. Carotenoids were identified by comparison to standard retention times and UV-visible spectral peaks. The quantification of carotenoids was performed using the standard curve method, and the concentration of carotenoids was expressed as μg/g fresh weight (FW). All carotenoid standards including β-carotene, α-carotene, phytoene, β-cryptoxanthin, neoxanthin, lutein, and violaxanthin were purchased from Sigma-Aldrich. Three replicates were used for each sample.

### Aroma Volatile Apocarotenoid Extraction and Quantification

The AVA contents in flesh were analyzed by gas chromatography mass spectrometry (GC-MS) as previously described (Zheng et al., 2016). A solid-phase microextraction (SPME) needle with a 1-cm long fiber coated with 65 μm of polydimethylsiloxane, and divinybenzene (Supelco Inc., Bellefonte, PA, United States) was used for volatile extraction. The identification and quantification of volatiles was performed on an Agilent 6890N GC equipped with a flame ionization detector (FID) detector and a DB-WAX column (0.32 mm, 30 m, 0.25 μm, J&W Scientific, Folsom, CA, United States). All volatiles were quantified according to standard curves of authentic compounds. β-Ionone, dihydro-β-ionone, β-damascenone, and 6-methyl-5-hepten-2-one were obtained from Sigma (St. Louis, MO, United States). Extracts from three triplicate tissue samples were analyzed.

### RNA Extraction and cDNA Synthesis

Total RNA was isolated and extracted from fruit flesh using a Tiangen reagent kit as described previously (Shan et al., 2008). RNA quantity and quality were determined in a NanoDrop 2000 spectrophotometer and through denaturing agarose gel electrophoresis. The first strand of cDNA was synthesized from 4 μg of total RNA as a template according to the protocol of the SuperScript^TM^ III RT-PCR First-Strand Synthesis System (Invitrogen, CA, United States) and then used as a template to conduct real-time quantitative PCR.

### Real-Time Quantitative PCR Analysis

The specific primers for the eleven genes involved in carotenoid biosynthesis (*PSY*, *PDS*, *ZDS*, *CRTISO*, *LCYb*, *CHYb*, *CYP*, *ZEP*, *NCED*, *CCD1*, and *CCD4*) and two reference genes (26 S ribosome and Actin) (Supplementary Table 1) were designed on the basis of our RNA-Seq data (NCBI Sequence Read Archive: PRJNA506502). Gene transcript levels were detected using an iQ5 instrument (Bio-Rad Laboratories, Inc., United States) with the SYBR Premix Ex Taq II Kit [TaKaRa Biotechnology (Dalian) Co., Ltd., China]. The amplification procedure was as follows: 95°C for 1 min, followed by 40 cycles at 95°C for 20 s, 58°C for 20 s, and 72°C for 30 s. The Ct values of the reactions were recorded, and gene transcript quantification was performed based on the relative expression of the selected genes against that of Actin using the 2^–ΔΔ*CT*^ method (Zhang et al., 2017). Three biological replicates were used for each analysis.

### Statistical Analysis

Data were expressed as the mean of three biological replicates ± standard deviation (SD) using Microsoft Excel 2010. Statistical differences for each treatment point were tested by Fisher’s protected least squares difference (LSD) test at a 0.01 probability. The correlation analysis was conducted by RStudio (Version 1.1.463). All graphs were drawn with Origin Pro 2018 (OriginLab Corporation, Northampton, MA, United States).

## Results

### Evaluation of Fruit Ripening According to Basic Fruit Quality Parameters

**FIGURE 1:**
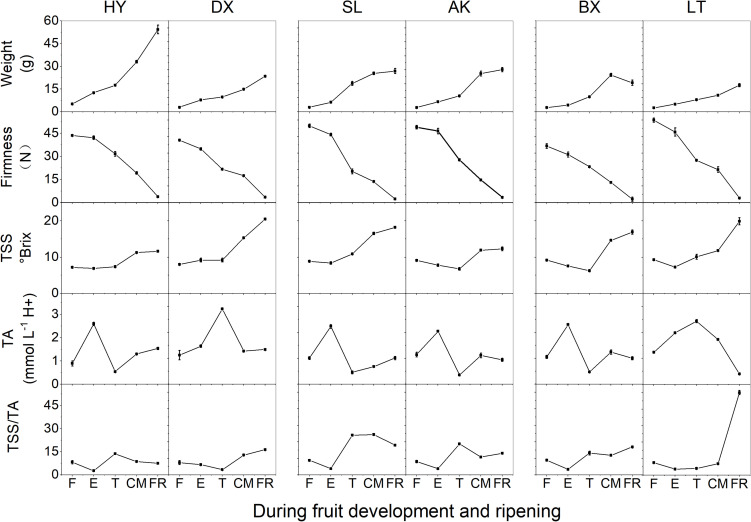
Basic quality parameters of apricots during development and ripening. TA, titratable acid; TSS, total soluble solid; TSS/TA, the ratio of TSS and TA. F, E, T, CM, and FR represent fruitlet, enlargement, turning, commercial maturation, and fully ripe developmental stages of fruit, respectively. All data are expressed as the means ± standard deviations of three biological replicates.

The changes in basic fruit quality parameters for the six apricot cultivars of three skin types are shown in Figure 1. During fruit development and ripening, the fruit weight of all cultivars increased significantly (*p* < 0.01). Although the TSS content remained stable from the F to E stages in all cultivars, it increased rapidly from the T to FR stages, and similar changes were observed for TSS/TA. Firmness decreased markedly throughout the development and ripening period (*p* < 0.01). The TA contents of the six cultivars increased to the maximum at the E or T stage and then decreased in the later stage of fruit development. The changes in fruit quality parameters suggested that the early stages, F and E, correspond to development, while the late stages, T, CM, and FR, correspond to ripening.

### Color Parameters of Different Cultivars During Fruit Development and Ripening

**FIGURE 2:**
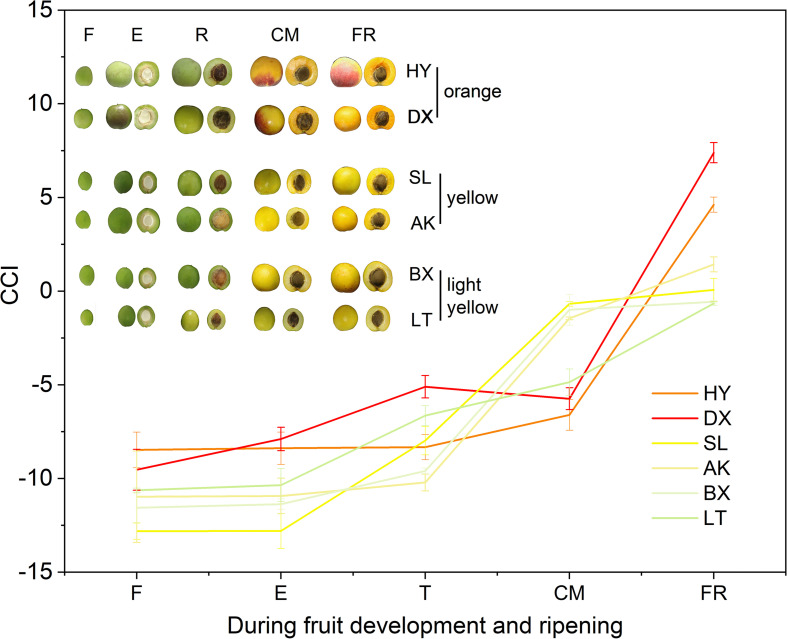
Citrus color index (CCI) values of three types of cultivars during fruit development and ripening. F, E, T, CM, and FR represent fruitlet, enlargement, turning, commercial maturation, and fully ripe developmental stages of fruit. All data are expressed as the means ± standard deviations of three biological replicates.

The peel color of the fruits changed dramatically throughout the fruit development period (*p* < 0.01). According to the peel color at the fully ripe (FR) stage, the six apricot cultivars could be clearly divided into three skin color types: orange, yellow, and light-yellow (Figure 2). With the development of fruit, the values of L^∗^, a^∗^, b^∗^, and c^∗^ continuously increased (Supplementary Table 2), indicating that the fruit peel color gradually changed from green to yellow. Notably, the hue values (h) of the six apricot cultivars at the F stage were basically the same, ranging from 114.91 ± 0.59 to 125.31 ± 6.38 (Supplementary Table 2). At the FR stage, the *h* values of the two orange cultivars, “HY” and “DX,” decreased to 72.57 ± 0.40 and 67.36 ± 2.90, respectively, which were significantly lower than the *h* values of the two yellow cultivars, “SL” and “AK,” and much lower than those of the two light-yellow cultivars, “BX” and “LT,” respectively (Supplementary Table 2), which is completely consistent with the definition of the *h* value as representing reddish-purple at 0°, yellow at 90°, and bluish-green at 180°. In addition, the color differences between the cultivars were well described by the CCI values. At the FR stage, the six cultivars were divided into three types based on their CCI values, which was consistent with the peel color of each cultivar at the fully ripe stage (Figure 2).

### Carotenoid Profile of Each Cultivar During Fruit Development and Ripening

**FIGURE 3:**
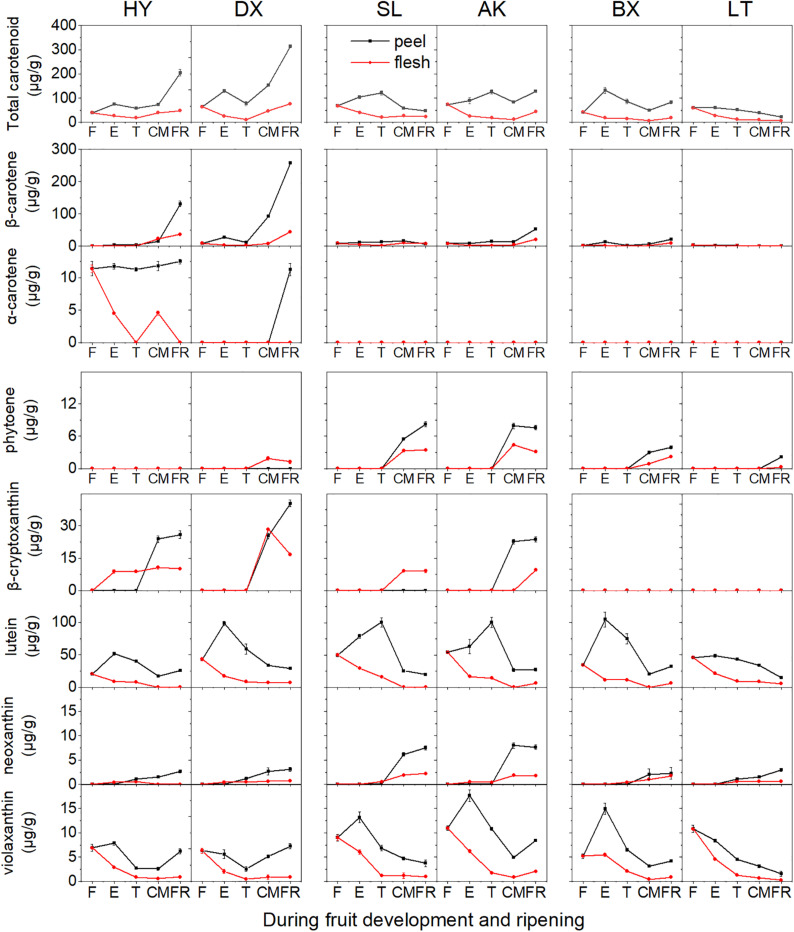
Carotenoid content (μg/g fresh weight) of apricot fruit during development and ripening. F, E, T, CM, and FR represent fruitlet, enlargement, turning, commercial maturation, and fully ripe developmental stages of fruit. All data are expressed as the means ± standard deviations of three biological replicates.

In total, seven carotenoids were identified from the tested apricots, including β-carotene, α-carotene, phytoene, β-cryptoxanthin, neoxanthin, lutein, and violaxanthin. Among these carotenoids, β-carotene was predominant in the fruits of all apricot cultivars, followed by lutein. The total carotenoid content in the peel was markedly higher than that in the flesh despite fruit development (Figure 3 and Supplementary Figures 1, 2).

The total carotenoid content continuously increased in the orange cultivars “HY” and “DX” throughout the development period, while it decreased during fruit ripening in the yellow (“SL” and “AK”) and light-yellow (“BX” and “LT”) cultivars (Figure 3). The contents of five of the detected carotenoids (β-carotene, α-carotene, phytoene, β-cryptoxanthin, and neoxanthin) generally increased in all six cultivars throughout the fruit development period, whereas those of the other two carotenoids (lutein and violaxanthin) continued to increase until the E or T stage and then decreased until the FR stage in the peel and continued to decrease throughout the remaining development stages in the flesh.

The highest total carotenoid content was observed in the orange cultivars, while the total carotenoid contents detected in the yellow and light-yellow cultivars were only 27–63% and 19–20%, respectively, of those in the orange cultivars. It was notable that the composition and content of carotenoids differed in the cultivars. In the orange cultivars, only phytoene was not detected in “HY,” while “DX” showed enrichment of all carotenoids identified. The contents of β-carotene, β-cryptoxanthin, and specific α-carotene in “HY” and “DX” were markedly higher than those in the yellow and light-yellow cultivars (*p* < 0.01). Conversely, although the content of phytoene increased in all cultivars throughout the development period, the lowest content was observed in the orange cultivars. In the yellow cultivars, α-carotene was not detected, and higher contents of neoxanthin and violaxanthin were detected than in the orange and light-yellow cultivars. The content of β-carotene was significantly lower than that in the orange cultivars (*p* < 0.01). In the light-yellow cultivars, α-carotene, and β-cryptoxanthin were not detected, and the content of β-carotene was lower than that in any of the other cultivars included in this study.

**FIGURE 4:**
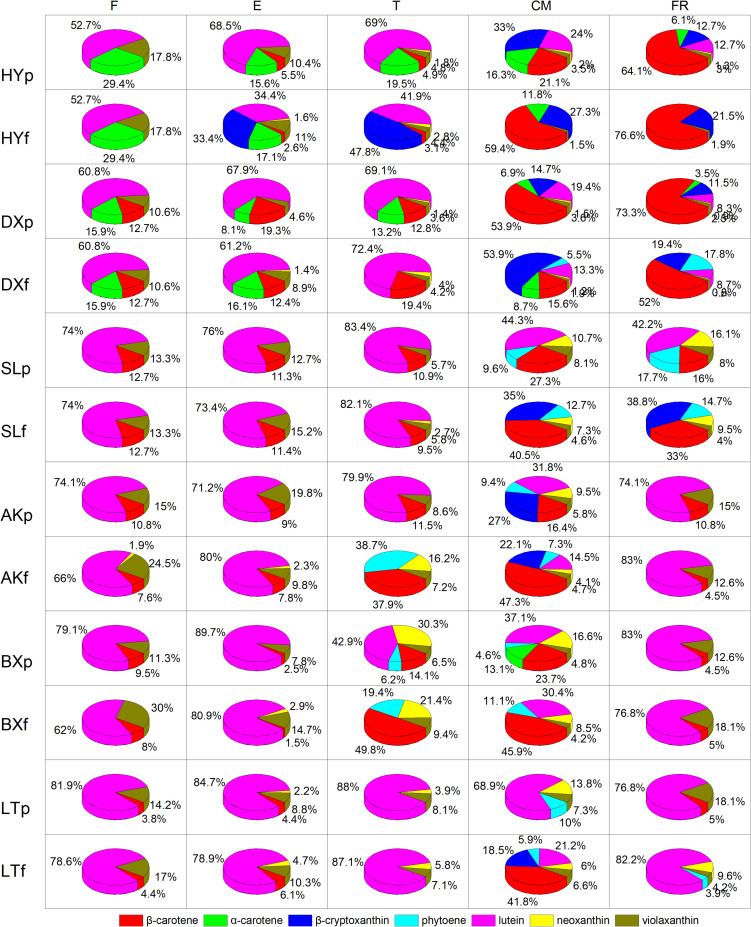
Proportion of carotenoids during fruit development and ripening. p and f represent the peel and flesh, respectively. βca, β-carotene; αca, α-carotene; βcr, β-cryptoxanthin; phy, phytoene; lut, lutein; neo, neoxanthin; vio, violaxanthin. F, E, T, CM, and FR represent five developmental stages of fruit. All data are expressed as the means ± standard deviations of three biological replicate.

The proportions of the carotenoids varied significantly between fruits of different stages in each cultivar (Figure 4). Lutein was the predominant carotenoid in all cultivars in the early development period (from F to T), accounting for 52.7–89.7% of the total carotenoids in peels and 34.4–83% of the total carotenoids in flesh. In contrast, the proportion of lutein decreased dramatically during ripening (from T to FR), accounting for 8.3–44.3% of the total carotenoids in peels and 0–30.4% of the total carotenoids in flesh, and this carotenoid was replaced by increasing amounts of β-carotene and β-cryptoxanthin. During the ripening period, the proportion of β-carotene in flesh increased to 15.6–76.6% in the orange cultivars, 33–47.3% in the yellow cultivars, and 45.9–49.8% in the light-yellow cultivar “BX,” but this carotenoid did not accumulate in “LT,” and the proportion of β-cryptoxanthin in flesh increased to 19.4–53.9% in the orange cultivars and no detected (ND) to 38.8% in the yellow cultivars, but this carotenoid did not accumulate in either light-yellow cultivar. Moreover, at the FR stage, the carotenoids in the flesh of “HY” and “DX” were dominated by β-carotene, which is a natural pigment conferring an attractive orange color, accounting for 76.6% and 52% of the total carotenoids, respectively. The flesh of “SL” and “AK” was dominated by β-cryptoxanthin and β-carotene, respectively, accounting for 38.8% and 47.3% of the total carotenoids. The flesh of “BX” and “LT” was dominated by β-carotene and lutein, respectively, accounting for 45.9% and 82.2% of the total carotenoids.

### Aroma Volatile Apocarotenoid Profile of Each Cultivar During Fruit Development and Ripening

**FIGURE 5:**
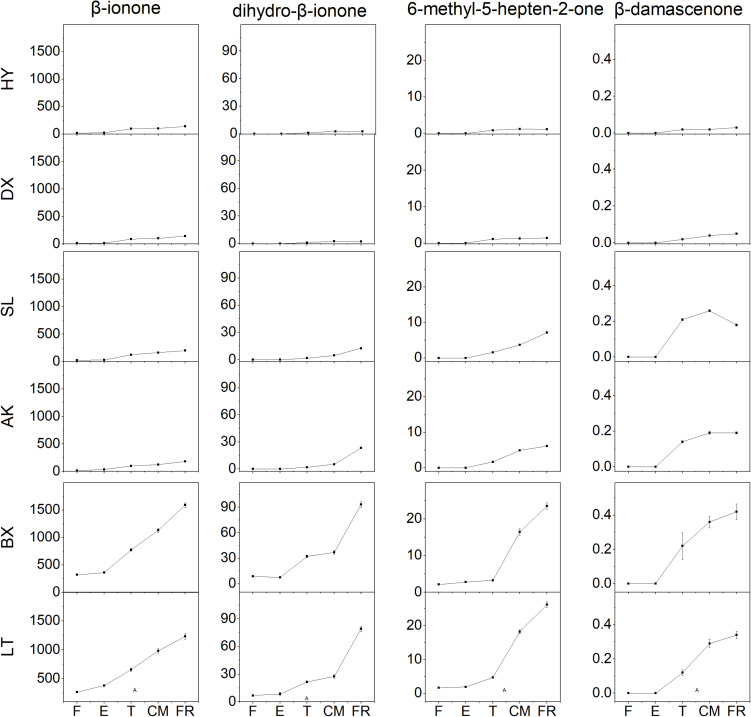
Aroma volatile apocarotenoid content of apricot fruit development and ripening. F, E, T, CM, and FR represent fruitlet, enlargement, turning, commercial maturation, and fully ripe developmental stages of fruit. All data are expressed as the means ± standard deviations of three biological replicates.

Four aroma volatile apocarotenoids were identified from the fruit of cultivars tested, including β-ionone, dihydro-β-ionone, 6-methyl-hepten-2-one, and β-damascenone (Figure 5). β-ionone was the predominant component and its content increased rapidly from the T stage in all yellow and light-yellow cultivars, but it remained very low level in orange cultivar throughout ripening process. Similar changes dihydro-β-ionone, 6-methyl-hepten-2-one, and β-damascenone were observed these varieties during ripening. Even increased four aroma volatile apocarotenoids were also detected in yellow cultivars “SL” and “AK”; however, their abundance of these compounds in light-yellow cultivars “BX” and “LT” were considerably higher than those in yellow cultivars during ripening.

### Differential Transcription Levels of Genes Involved in Carotenoid Biosynthesis

**FIGURE 6:**
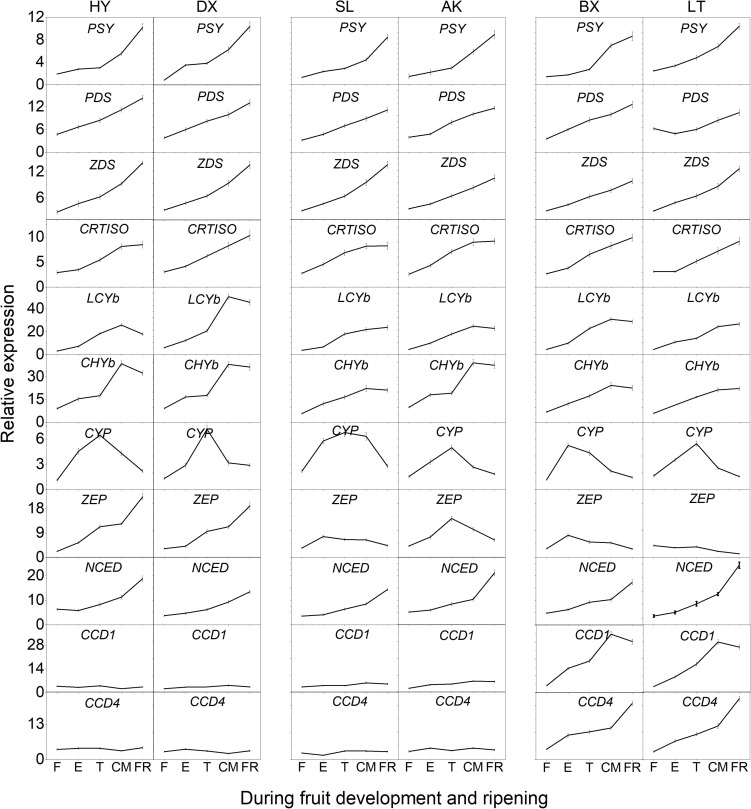
Expression pattern of genes involved in carotenoid biosynthesis during fruit development and ripening. F, E, T, CM, and FR represent fruitlet, enlargement, turning, commercial maturation, and fully ripe developmental stages of fruit. PSY, phytoene synthase; PDS, phytoene desaturase; Z-ISO, ζ-carotene isomerase; ZDS, ζ-carotene desaturase; CRTISO, carotenoid isomerase; LCYe, lycopene ε-cyclase; LCYb, lycopene β-cyclase; CHYb, β-carotene hydroxylase; CYP, cytochrome P450-type monooxygenase 97C; ZEP, zeaxanthin epoxidase; VDE, violaxanthin de-epoxidase; NXS, neoxanthin synthase; CCD, carotenoid cleavage dioxygenase; NCED, 9-*cis-*epoxycarotenoid dioxygenase. All data are expressed as the means ± standard deviations of three biological replicates.

The expression of 11 genes involved in carotenoid biosynthesis was analyzed during the entire developmental period (Figure 6). Among these genes, the transcript levels of six genes (*PSY*, *PDS*, *ZDS*, *CRTISO*, *LCYb*, and *CHYb*) continued to increase as the fruits matured and generally showed the highest transcript levels in the orange cultivars, which corresponded to the highest levels of phytoene, β-carotene, α-carotene, and β-cryptoxanthin found in orange flesh. It is worth noting that the expression of *CHYb* in the yellow cultivars was significantly increased during ripening (*p* < 0.01), which might have resulted in the simultaneous increase in the proportion of β-cryptoxanthin from ND to 38.8%. In addition, the expression of *CYP* continued to increase during the early development stages (from F to E) then decreased until the FR stage in all six cultivars, corresponding to the decline in lutein content. The transcript level of *ZEP* in the yellow and light-yellow cultivars was similar to that of *CYP*, which was consistent with the decrease in the violaxanthin content. Although the expression of *ZEP* increased in the orange cultivars, the content of violaxanthin decreased unexpectedly as the fruit matured, which might have been due to the increased transcript level of *NCED*, whose product converts violaxanthin into abscisic acid. In addition, the transcript levels of *NCED*, *CCD1*, and *CCD4* continued to increase throughout the fruit development period were highest in the light-yellow cultivars, which could result in more carotenoids in “BX” and “LT” being converted into apocarotenoids, leading to lower carotenoid levels.

### Correlation Between Carotenoid Contents, Aroma Volatile Apocarotenoid Contents and Expression Levels of Carotenogenic Genes

**FIGURE 7:**
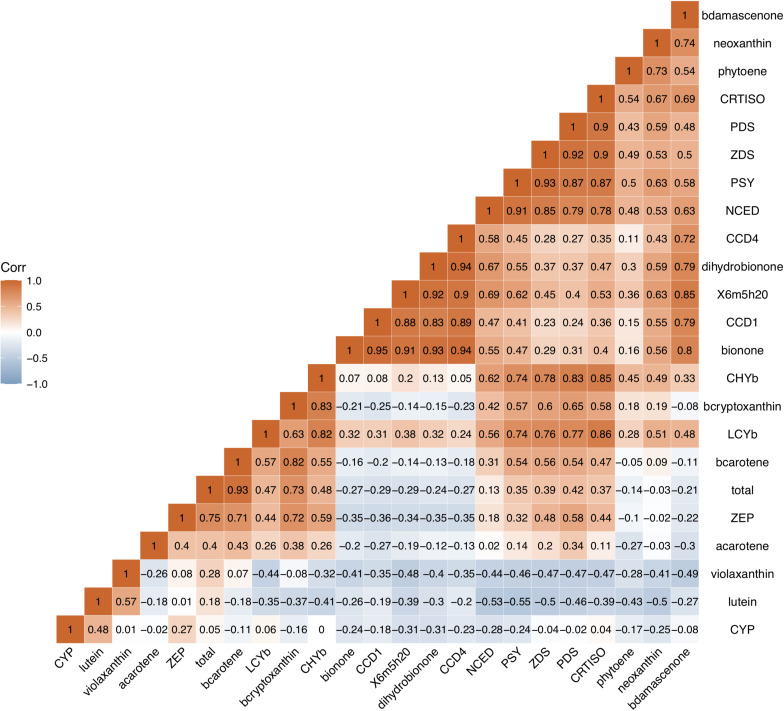
Correlation analysis between carotenoid contents, aroma volatile apocarotenoid contents and expression levels of carotenogenic genes. b -ionone, dihydroionone, X6m5h20, bdamascenone, total, bcarotene, acarotene, and bcryptoxanthin, represent β-ionone, dihydro-β-ionone, 6-methyl-hepten-2-one, β-damascenone, total, bcarotene, acarotene, and bcryptoxanthin total carotenoid, β-carotene, α-carotene, and β-cryptoxanthin, respectively. These genes are same as them in Figure 6.

Correlation analysis was used to investigate the relationship between carotenoid contents, aroma volatile apocarotenoid contents and expression levels of genes in carotenoid biosynthesis pathway (Figure 7). A high positive correlation between the total carotenoid, β-carotene, β-cryptoxanthin contents, and expression level of *ZEP* in all cultivars studied was observed (*p* < 0.01, *r* > 0.71), high correlation was also found between β-carotene content and expression level of *CHYb*. Significant negative correlation was found between carotenoids accumulation and expression levels of *CCD1* or *CCD4*. Significant positive correlation was observed between four AVAs (β-ionone, dihydro-β-ionone, 6-methyl-hepten-2-one, and β-damascenone) contents and the expressions of *CCD1* and *CCD4* (*p* < 0.01, *r* ranged 0.72–0.95), while no significant correlation was found between the expressions of *NCED* and AVAs content (*p* < 0.01, *r* < 0.67).

## Discussion

In flowering plants, the composition and accumulation of carotenoids cause many fruits to exhibit different colors, such as yellow, orange or red (Sagawa et al., 2016). An increasing number of studies have shown that the color differences in the fruits of different cultivars are closely related to the composition and content of carotenoids and the transcript levels of key genes in the carotenoid metabolism pathway (Qi et al., 2019). The specific reason for the color difference in apricot cultivars is unknown. In the present study, we first explored the different mechanisms of carotenoid accumulation in three skin types of apricot cultivars during fruit development and ripening.

### Three Skin Types of Apricots Present Different Carotenoid and AVA Profile

Previous studies have shown that the color change of fruit is closely associated with the contents and proportions of carotenoids during development and ripening (Yuan et al., 2015). Here, the sharply contrasting peel colors of the three skin types of apricot cultivars indicated that there was a significant difference in the accumulation of carotenoids. The total carotenoid contents of the orange cultivars were much higher than those of the yellow and light-yellow cultivars, suggesting that the degree of fruit coloration may depend on the content of total carotenoids. The contents and proportions of β-carotene and β-cryptoxanthin increased significantly, and these compounds became the main pigments during the ripening of the orange cultivars, which explains why these cultivars show a vivid orange color when their fruits are fully ripe; these results are consistent with findings for the same color type of tomato and carrot (Rodriguez-Concepcion and Stange, 2013), but differ from findings for pepper (Ha et al., 2007) and papaya (Schweiggert et al., 2011). The increase in the content of β-cryptoxanthin was accompanied by a decrease in the content of lutein, indicating that the β-branch was strengthened in the ripening orange and yellow cultivars. However, β-cryptoxanthin was not detected in the light-yellow cultivars, suggesting that the carotenoid biosynthetic pathway in the light-yellow cultivars only involved the α-branch. The content of lutein was greatly reduced, making lutein the major pigment in the light-yellow cultivars. In contrast, lutein is the main pigment of most yellow cultivars, including pepper and *Gentiana lutea* (Zhu et al., 2014). On the whole, orange and yellow skin cultivars accumulated rich both the carotenoid composition and the total carotenoid content, these cultivars belong to the “carotenoid accumulation” cultivars during fruit development and ripening, but light-yellow skin cultivars decreased the carotenoid throughout the period, they can be as “carotenoid loss” cultivars. On the contrary, “carotenoid loss” cultivars produced rich AVAs, but “carotenoid accumulation” cultivars not. The fluctuation of carotenoids and AVAs in different skin types apricot cultivars suggested that there is a putative link between carotenoid accumulation and AVA produce in different skin types of apricot cultivars.

### Carotenoid Accumulation Highly Correlated With the Transcript Level of Carotenoid Biosynthesis Related Genes

Our mRNA transcript level analysis showed that the transcript dynamics of genes involved in carotenoid biosynthesis could explain the differences in carotenoid accumulation patterns in cultivars with different skin types. For example, a low carotenoid content of loquat fruit is correlated with lower mRNA transcript levels of *PSY1*, *CYCB*, and *BCH* (Fu et al., 2012; Hadjipieri et al., 2017). Phytoene synthase (PSY) is the core determinant of the total amount of carotenoid accumulation in plant tissues (Burkhardt et al., 2010), this enzyme produces phytoene, providing the synthetic precursor for other carotenoids. Previous studies have shown that upregulation of *PSY* in watermelon promotes carotenoid accumulation (Lv et al., 2015). In the present study, we found that the transcript patterns of *PSY* were not significantly different among the three cultivars, but the relative transcription level of the *PSY* gene increased significantly, accompanied by carotenoid accumulation during apricot fruit development and ripening, suggesting that *PSY* plays an important regulatory role in carotenoid biosynthesis and accumulation. Phytoene desaturase (PDS) and ζ-carotene desaturase (ZDS) are responsible for the synthesis of lycopene from phytoene, and mutations in the *PDS* and *ZDS* genes result in the accumulation of phytoene and ζ-carotene in maize and *Arabidopsis*, respectively (Dong et al., 2007). Here, we found that phytoene was not predominant during ripening in the three skin types of apricot cultivars, suggesting no significant correlation between carotenoid accumulation and the expression of the *PDS* and *ZDS* genes, and these two genes play relatively minor roles in apricot carotenoid accumulation during ripening. *Arabidopsis*, tomato and melon mutants of carotenoid isomerase (CRTISO) accumulate *cis*-carotenes in the etioplasts of seedlings or chromoplasts of the fruit (Galpaz et al., 2013). We found that the transcript level of *CRTISO* increased significantly and promoted the accumulation of β-carotene during the process of fruit development and ripening. Lycopene β-cyclase (LCYb) allows lycopene to synthesize β-carotene (Alquézar et al., 2009), and a high transcript level of *LCYb* contributes to the accumulation of β-carotene. The accumulation pattern of β-carotene in different kiwifruit cultivars is related to the expression of the *LCYb* gene (Ampomah-Dwamena et al., 2009). The content of β-carotene in the fruit was found to be increased 3.8 times after the *LCYb* gene was introduced into the tomato (Rosati et al., 2010). In this work, higher expression of *LYCb* was accompanied by β-carotene accumulation in the three cultivars. β-carotene hydroxylase (CHYb) catalyzes the conversion of β-carotene to β-cryptoxanthin and zeaxanthin, and the expression of *CHYb* results in higher accumulation of β-cryptoxanthin in the orange and yellow cultivars than in the light-yellow cultivars. The transcript level of the ε-carotene hydroxylase gene *CYP97* (*CYP*) is positively correlated with lutein production in the carotenoid biosynthesis pathway (Yuan et al., 2015), and it has been shown that the *Arabidopsis* lut5 mutant, which is defective in CYP97A3 hydroxylase, accumulates higher levels of α-carotene (Kim and DellaPenna, 2006). The expression level of *CYP* and the content of lutein first increased and then decreased during development and ripening in the three examined skin types of apricot cultivars. Zeaxanthin epoxidase (ZEP) is an enzyme in the xanthophyll cycle, and ZEP catalyzes the conversion of zeaxanthin to violaxanthin. Loss of *ZEP* function in the aba1 mutant of *Arabidopsis* and the aba2 mutant of tobacco causes the accumulation of high zeaxanthin levels in leaves and lower ABA levels (Marin et al., 1996). In this study, the change in the content of violaxanthin was consistent with the change in the *ZEP* gene transcript level, indicating that the *ZEP* gene positively regulates the accumulation of violaxanthin in the carotenoid biosynthesis pathway.

### Two Carotenoid Cleavage Dioxygenase Genes Play an Important Role in Coloration and Aroma Formation of Apricots

Apocarotenoids are formed by the enzymatic cleavage of carotenoids with the assistance of carotenoid cleavage dioxygenase (CCD) family proteins (Figure 1; Walter and Strack, 2011), which are non-heme iron (II) dependent enzymes (Harrison and Bugg, 2014). In plants, CCDs generally include 9-*cis-*epoxycarotenoid dioxygenase (NCED), CCD7, CCD8, CCD4, and CCD1 (Walter et al., 2010). NCED enzymes specifically cleave double bond at the 11,12 position of 9-*cis-*epoxycarotenoids, resulting in the production of *cis*-xanthoxin, an abscisic acid (ABA) precursor (Schwartz et al., 2001). AtCCD7 catalyzes the asymmetric cleavage of β-carotene at the 9′,10′ position, producing 10′-apo-β-caroten-10′-al and β-ionone, while 10′-apo-β-caroten-10′-al is subsequently cleaved by CCD8, yielding strigolactones (SLs) (Seto and Yamaguchi, 2014). AtCCD4, a negative regulator of the carotenoid accumulation in Arab. thaliana seeds, cleaves C9–C10 double bond of carotenoids (Bruno et al., 2016). The CCD4 protein cleaves β-cryptoxanthin and zeaxanthin at the 7,8 or 7′,8′ position to form β-citraurin, thereby influencing color formation in citrus (Ma et al., 2013). Previous studies found that the emission of β-ionone from *Petunia hybrida* flowers correlated strongly with the expression levels of *PhCCD1* in corollas (Simkin et al., 2004). In strawberry, the expression of *CCD1* reduces the content of lutein (García-Limones et al., 2008). Studies have shown that *CCD4* can control the pigmentation of peach flesh, and high *CCD4* transcript abundance in white flesh is related to the release of carotenoid-derived volatiles (Falchi et al., 2013). During apricot fruit development and ripening, we found that the CCDs gene family members *CCD1* and *CCD4* showed lower transcript levels in the orange and yellow cultivars but higher transcript levels in the light-yellow cultivars. The light-yellow cultivars exhibit the lowest total carotenoid and β-carotene contents but the highest transcript levels of the corresponding genes among the three skin types of cultivars. By contrast, the orange cultivar shows the lowest transcript levels and the highest total carotenoid and β-carotene contents. Simultaneously, the richest contents of aroma volatile apocarotenoids were detected in light-yellow fruit, their contents were almost undetected in orange cultivars, and the contents positively correlated significantly with the expression levels of *CCD1* and *CCD4*, but correlated with negatively total carotenoid accumulation. Furthermore, we compared the DNA sequences of *CCD1* and *CCD4* from the six experimental cultivars found that these sequences are the same, showing that expression levels of *CCD1* and *CCD4* is important for carotenoid accumulation in these cultivar fruit. These findings suggest that the total carotenoid and β-carotene contents in apricot fruit are negatively regulated by *CCD1* and *CCD4*.

## Conclusion

The total carotenoid content continuously increased in the orange cultivars during ripening, while it decreased in the light-yellow cultivars, and the orange cultivars presented the highest levels of total carotenoids, followed by the yellow cultivars. The composition and contents of carotenoids differed in the cultivars. High levels of β-carotene and specific α-carotene contributed greatly to the coloration of the orange cultivars, but the yellow cultivars were characterized by high levels of oxidated carotenoids and phytoene, low levels of β-carotene, an absence of β-carotene and β-cryptoxanthin and decreased levels of oxidated carotenoids, resulting in light-yellow coloration. Based on the accumulation patterns of carotenoids in different apricot cultivars during development and ripening, the apricot cultivars can be divided into two types: “carotenoid accumulation” and “carotenoid loss” types of cultivars. In contrast, “carotenoid loss” types of cultivars fruit were characterized by rich AVAs such as β-ionone. The differences in carotenoid and AVAs accumulation between the cultivars were coordinately determined by the differential expression of carotenoid biosynthetic and cleaving genes, especially *PSY*, *PDS*, *ZDS*, *LCYb*, *ZEP*, *NCED*, *CCD1*, and *CCD4*. However, it is necessary to further investigate how these enzymes regulates carotenoids and AVAs accumulation through substrate specific identification *in vivo* and *in vitro* and transgenic means to verify the role played by their enzyme. This study unveils the chemical mechanism of color and aroma of different skin types of apricot cultivars, provides useful targets for controlling fruit quality in future work.

## Data Availability Statement

The original contributions presented in the study are included in the article/Supplementary Material, further inquiries can be directed to the corresponding author.

## Author Contributions

GZ designed the experiments. WX performed the experiments and wrote the manuscript. LZ and SL analyzed the data. All authors contributed to the article and approved the submitted version.

## Conflict of Interest

The authors declare that the research was conducted in the absence of any commercial or financial relationships that could be construed as a potential conflict of interest.

## Supplementary Material

Supplementary Figure 1 HPLC chromatogram of carotenoids from the peel of “Danxing” at the fully ripe stage.

Supplementary Figure 2 HPLC chromatogram of carotenoids from the peel of “Baixing” during development and ripening. The black line, blue line, green line, indigo line, and pink line represent the fruitlet (F), enlargement (E), turning (T), commercial maturation (CM), and fully ripe (FR) stages.

Supplementary Table 1 Primer sequences for qRT-PCR.

Supplementary Table 2 Changes in chromatism parameters of apricot fruit during development and ripening.
