# Supplementary material for: The Genes of CYP, ZEP, and CCD1/4 Play an Important Role in Controlling Carotenoid and Aroma Volatile Apocarotenoid Accumulation of Apricot Fruit
Source: Front Plant Sci. 2020 Dec 18;11:607715. doi: 10.3389/fpls.2020.607715 (PMC7775601; doi:10.3389/fpls.2020.607715)
Supplement: Supplementary Figure 1 — HPLC chromatogram of carotenoids from the peel of “Danxing” at the fully ripe stage. [file Table_1.DOCX]

Supplementary Material

**Table S1** Primer sequences for qRT-PCR.

| Primer | Forward primer（5′ to 3′） | Reverse primer（5′ to 3′） |
| --- | --- | --- |
| *26 S* | GATAACGCAGGTGTCCTAAGATGA | ATTCCGAAGGTCTAAAGGATCGA |
| *ACT* | GTTATTCTTCATCGtCGTCTTCG | CTTCACCATTCCAGTTCCATTGTC |
| *PSY* | CCTCTACTGCTACTATGTTGCTGG | CATTTTCCACTTGTCAGTCACCT |
| *PDS* | GGGTCGTTTCCAGTGGCTAA | GAAAACAACCCAAGACATAGAGC |
| *ZDS* | TGGAAGGGGCAACTTTGTCTGG | TTCTTTCGCAATCCCACCAACTC |
| *CRTISO* | TGCGGGGACATCTTTACCA | GAGCCAGGCACCATATCACTT |
| *LCYb* | GCTTGATCTGGATGGGAGTAGA | TTTAACCAAAGGCAAAGGACAC |
| *CHYb* | GTTGAACCCGAAAAGCCAATA | TTCTTCCTCTTCTTCTTCCACCT |
| *CYP* | AGACTACTTCCGTTTGGAGCCG | CCTTTGGATTCATCCCTTCAGC |
| *ZEP* | AAGGATGGAGGCAAGTGGG | CGATGTAGCTCCGTGGACAGT |
| *NCED* | TCAACCACCCTTTTCACTCCCT | GGCACTGGCTTTGAGGATTTAG |
| *CCD1* | CTCGTGTTCCCGGAGTTACC | CGACCGGGTCAGATGACATT |
| *CCD4* | CCACCTCCACAAAAGCACCA | TGCAGGTAGCGTAGGATCTGT |

**Table S2** Changes in chromatism parameters of apricot fruit during development and ripening.

| Cultivars | Stage | L^*^±SD^b^ | a^*^±SD | b^*^±SD | c^* c^±SD | h ^d^±SD |
| --- | --- | --- | --- | --- | --- | --- |
|  |  |  |  |  |  |  |
| HY | F | 54.97±0.97 | -16.04±0.71 | 34.44±0.77 | 37.99±0.94 | 114.91±0.59 |
|  | E | 55.53±0.87 | -15.08±1.26 | 32.41±1.68 | 33.91±0.59 | 115.17±0.16 |
|  | T | 56.13±0.62 | -14.46±0.18 | 30.95±0.43 | 35.35±0.72 | 112.05±0.54 |
|  | CM | 61.53±0.33 | -13.30±0.53 | 32.74±0.57 | 52.69±13.79 | 72.65±11.25 |
|  | FR | 65.11±0.99 | 12.78±0.29 | 42.51±1.44 | 45.14±1.29 | 72.57±0.40 |
| DX | F | 54.52±0.34 | -20.28±0.26 | 39.02±0.70 | 43.98±0.63 | 117.46±0.53 |
|  | E | 57.41±0.31 | -16.87±3.16 | 37.23±0.38 | 41.78±0.45 | 116.97±0.21 |
|  | T | 63.51±0.84 | -12.31±2.86 | 37.98±1.31 | 44.35±0.39 | 110.43±0.64 |
|  | CM | 65.02±0.32 | -15.51±0.47 | 41.54±0.41 | 42.26±1.04 | 76.26±2.02 |
|  | FR | 55.49±3.42 | 17.97±1.93 | 43.83±1.05 | 47.67±0.52 | 67.36±2.90 |
| SL | F | 47.04±0.57 | -20.71±0.24 | 34.38±0.97 | 40.37±0.73 | 125.31±6.38 |
|  | E | 47.68±0.14 | -19.58±0.17 | 32.09±0.31 | 37.61±0.36 | 121.45±0.01 |
|  | T | 57.11±1.08 | -17.10±0.78 | 37.59±0.53 | 41.34±0.44 | 114.44±1.19 |
|  | CM | 68.35±0.48 | -2.35±0.73 | 51.09±1.35 | 51.41±1.25 | 92.70±0.08 |
|  | FR | 61.93±1.87 | 0.20±1.96 | 44.89±1.70 | 45.00±1.66 | 89.98±2.19 |
| AK | F | 50.02±0.63 | -21.98±0.20 | 40.07±0.13 | 45.56±0.17 | 118.79±0.19 |
|  | E | 50.90±0.16 | -21.76±0.20 | 39.12±0.18 | 44.77±0.25 | 119.15±0.10 |
|  | T | 52.19±0.45 | -19.02±0.19 | 35.71±0.94 | 40.47±0.92 | 118.11±0.40 |
|  | CM | 68.35±0.84 | -4.97±0.90 | 50.10±1.00 | 50.44±0.89 | 95.76±1.08 |
|  | FR | 60.28±1.04 | 4.04±1.12 | 47.02±2.70 | 45.30±1.50 | 84.25±1.50 |
| BX | F | 48.58±0.70 | -21.23±0.51 | 37.80±0.94 | 43.35±1.06 | 119.33±0.04 |
|  | E | 50.91±0.87 | -21.4±0.21 | 36.98±0.84 | 42.72±0.83 | 120.10±0.35 |
|  | T | 54.63±0.82 | -19.24±0.20 | 36.72±0.98 | 41.47±0.95 | 118.30±0.36 |
|  | CM | 67.10±0.59 | -3.06±1.93 | 45.86±1.16 | 46.12±1.17 | 93.71±1.17 |
|  | FR | 63.17±0.99 | -1.58±1.72 | 44.36±1.04 | 44.81±0.87 | 91.69±2.27 |
| LT | F | 49.19±2.11 | -19.84±0.64 | 37.99±2.64 | 42.95±2.68 | 117.95±0.96 |
|  | E | 51.69±0.70 | -20.76±0.24 | 38.80±1.19 | 43.55±0.46 | 118.57±0.37 |
|  | T | 59.03±2.35 | -15.62±0.76 | 39.86±0.62 | 42.90±0.35 | 111.59±1.10 |
|  | CM | 65.24±1.12 | -13.08±0.96 | 41.27±0.85 | 43.33±1.07 | 107.48±1.02 |
|  | FR | 66.26±0.68 | -1.86±1.03 | 43.30±0.66 | 43.37±0.69 | 92.56±1.22 |

^a^ Values with different letters showed statistically significant differences at the 5% level.

^b^ standard deviation

^c^ C^*^ = color intensity (chroma) ) = ( a^*2^ +b^*2^)^1/2^.

^d^ h = hue value = arctan ( b^*^/ a^*^).

**Figure S1** HPLC chromatogram of carotenoids from the peel of ‘Danxing’ at the fully ripe stage.

**
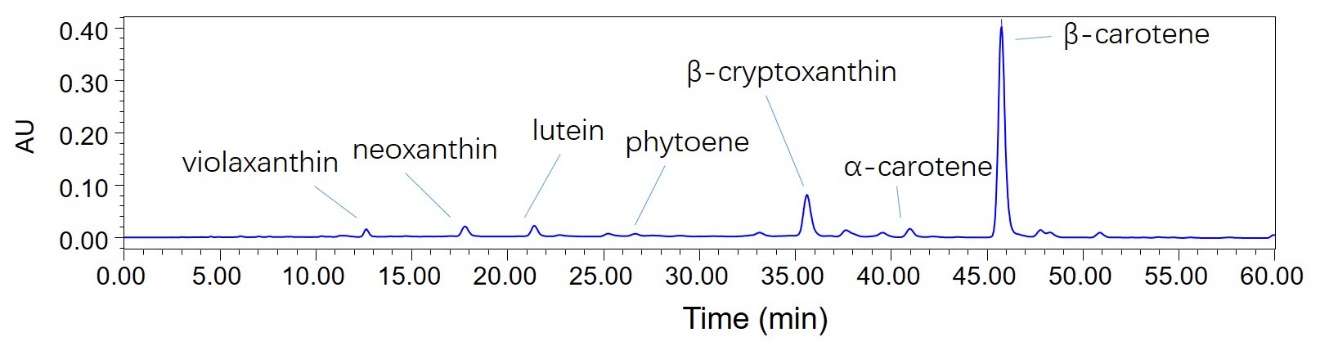
**

**Figure S2** HPLC chromatogram of carotenoids from the peel of ‘Baixing’ during development and ripening. The black line, blue line, green line, indigo line and pink line represent the fruitlet (F), enlargement (E), turning (T), commercial maturation (CM) and fully ripe (FR) stages.

**
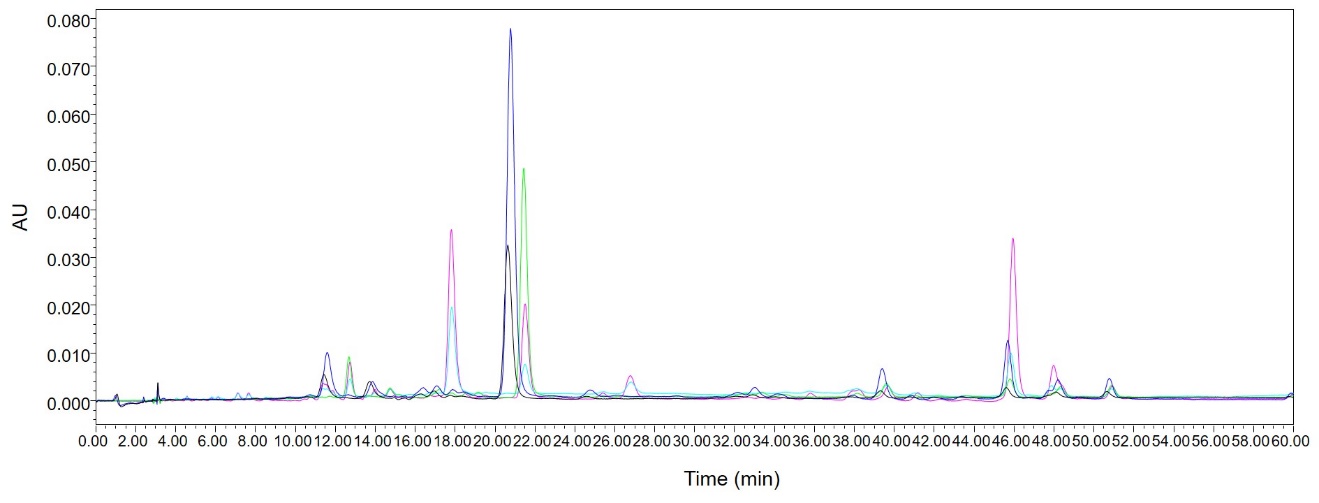
**
